# Evaluation of genotoxic effect via expression of DNA damage responsive gene induced by ivermectin on MDBK cell line

**DOI:** 10.1371/journal.pone.0296255

**Published:** 2024-05-03

**Authors:** Muhammad Muddassir Ali, Zainab Farhad, Muhammad Wasim, Sohail Raza, Mikhlid H. Almutairi, Kainat Zahra, Muhammad Usman Saleem, Khalid Mehmood

**Affiliations:** 1 Institute of Biochemistry and Biotechnology, University of Veterinary and Animal Sciences, Lahore, Pakistan; 2 Institute of Microbiology, University of Veterinary and Animal Sciences, Lahore, Pakistan; 3 Zoology Department, College of Science, King Saud University, Riyadh, Saudi Arabia; 4 Henry C. Lee Institute of Forensic Science, University of New Haven, West Haven, CT, United States of America; 5 Faculty of Veterinary Sciences, Department of Biosciences, Bahauddin Zakariya University, Bosan Road, Multan; 6 Faculty of Veterinary and Animal Sciences, Department of Clinical Medicine and Surgery, The Islamia University of Bahawalpur, Pakistan; University of Ibadan Faculty of Science, NIGERIA

## Abstract

Ivermectin (IVM) is an anti-parasitic drug which is used for treating parasitic infestations. It has been used in humans for treating intestinal strongyloidiasis and onchocerciasis however, currently researchers are investigating its potential for treating coronavirus SARS-CoV-2. Due to its broad-spectrum activities, IVM is being used excessively in animals which has generated an interest for researchers to investigate its toxic effects. Cytotoxic and genotoxic effects have been reported in animals due to excessive usage of IVM. Therefore, this study aims to evaluate the cytotoxic and genotoxic effects of IVM on the Madin-Darby-Bovine-Kidney (MDBK) cell line by examining the expression of a DNA damage-responsive gene (*OGG1*). Cytotoxicity of IVM was tested using an assay (MTT 3-(4,5-dimethylthiazol-2-yl)-2,5-diphenyltetrazolium bromide), whereas the genotoxicity was evaluated using comet assay along with micronucleus assay. Moreover, the gene expression of DNA damage response gene (*OGG1*) was measured by qRT-PCR, after extraction of RNA from the MDBK cell line using the TRIzol method and its conversion to cDNA by reverse-transcriptase PCR. During the experiment, cell viability percentage was measured at different doses of IVM i.e., 25%, 50%, 75%, along with LC50/2, LC50 and LC50*2. It was observed that the gene expression of *OGG1* increased as the concentration of IVM increased. It was concluded that IVM has both cytotoxic and genotoxic effects on the MDBK cell line. Furthermore, it is recommended that studies related to the toxic effects of IVM at molecular level and on other model organisms should be conducted to combat its hazardous effects.

## Introduction

Ivermectin (IVM) is a semi-synthetic macrocyclic compound which is effective against numerous parasitic diseases [1] including onchocerciasis. It has been reported to be effective against symptomatic alleviation caused by *Onchocerca volvulus* along with having the potential to reduce transmission of the aforementioned parasite [1].

The IVM exhibits systemic anti-parasitic activities against helminths, arachnids, insects [2] and is being used to combat parasitic issues related to animal and human health. It also provides positive effects to humans, livestock and pets however, its progressive impacts are still being investigated [3,4]. The IVM combat infections caused by many parasites and offers significant results for treating intestinal strongyloidiasis in humans. Currently being investigated as a potential treatment for COVID-19 (SARS-CoV-2) [5]. Moreover, due to its broad-spectrum effects IVM is being excessively used in both humans and animals [3].

Recently, IVM has gained significant attention due to the toxic effects caused by its excessive use. Slow metabolism of IVM in animals leads to its accumulation in tissues [6] and can be toxic to humans if they consume the meat of an animal that has been excessively treated with IVM [7].

Lower doses of IVM are tolerable for animals whereas, higher doses can cause hazardous effects. It has been reported that oral administration of IVM leads to significant reduction in microfilariae count in skin and ocular regions [8].

The FDA has approved usage of IVM in humans for treating various parasitic infestations. The IVM works by paralyzing the parasites that are causing infection. However, it has been scientifically proven that the excessive use of IVM leads to cell damage and genotoxicity [9]. When concentrations of IVM exceeds the recommended levels the capacity of P- glycoprotein pumps responsible for restricting the drug from entering the central nervous system (CNS) becomes overloaded resulting in neurotoxic disorders such as coma, encephalopathy, seizures, myoclonus, ataxia and tremors [10]. The results of (IVM 1.0%) have been studied on Chinese language hamster ovary (CHO(K1)) cells [11]. As stated above that IVM causes deleterious effects on both animals and humans therefore, the purpose of this study was to evaluate the cytotoxic and genotoxic effects of IVM by evaluating the expression of the DNA damage responsive gene (*OGG1*) in the Madin-Darby-Bovine-Kidney (MDBK) cell line.

## Materials and methods

### Cell line

For this study MDBK cell line was obtained from Department of Microbiology, University of Veterinary and Animal Sciences, Lahore, Pakistan. The DMEM (Dulbecco’s Modified Eagle Medium) media (Gibco DMEM, Thermo Fisher Scientific, USA) was used for MDBK cells.

Growth and sub-culturing of cell line

The MDBK cell line was sub-cultured by using already present cell line and DMEM media. Sub-culturing of the MDBK cell line was done by using frozen and fresh cell line stock. Frozen stock was thawed at 37°C in a water bath after which media was added and growth was obtained. Culture media was prepared using 3 mL antibiotic (Streptomycin) with 10 ml FBS (Fetal Bovine Serum) and DMEM was added up to 100 ml volume. Preceding media was removed from the flask and 3 mL for t25 and 6 ml for t75 trypsin was added for trypsinization (detachment of cells). Flasks were placed in an incubator for 3 min for detachment of cells. Media was added to stop trypsinization: 3 ml for t25 and 6 ml for the t75 flask. Centrifugation was done for 3 min. A pellet was obtained and new media was added for cell growth which was incubated for 24 hours at 37°C with 5% carbon dioxide.

### Cytotoxicity assay (MTT assay)

The MTT (MTT 3-(4,5-dimethylthiazol-2-yl)-2,5- diphenyltetrazolium bromide) was obtained in solid form with 0.5 mg/mL concentration. The MTT dye working solution was prepared as 1x PBS. Culture media was prepared by mixing antibiotics and FBS with DMEM in appropriate proportions. Cells were placed in a 96 well plate with a density of 1.5×10^3^ per well and 50 μl by volume and incubated at 37°C with 5% carbon dioxide for 24 hours. Media was removed after incubation and doses of ivermectin (1.25 μM, 2.5 μM, 5 μM, 10 μM, 20 μM, 40 μM, 80 μM, 160 μM, 320 μM, 640 μM) were given. A 50 μl dose was added to each well. Cells were incubated for 24 hours; media was removed, and MTT reagent was added at 0.5 mg/mL. Afterwards, 5 μl MTT reagent was added to each well and incubated for 3 hours. The solution was withdrawn and 100 μl DMSO was added to each well. Absorbance was measured at 560 nm by using an ELISA plate reader (Rayto Life and Analytical Sciences Co., Ltd. Shenzhen, China) [12].

### Genotoxicity assay (comet assay)

Comet assay was performed as described by previous study [13]. The MDBK cells were sub-cultured in a 6 well plate and treated with IVM at different doses (LD50, 2xLD50, LD50/2). Afterwards, the cells were isolated and mixed with low melting agarose (LMA) 0.8%, which was prepared by dissolving 0.008 g LMA in 1 mL of PBS. The cells were then placed on slides that were coated with normal melting agarose (NMA). For proper attachment of cells slides were placed on ice for 10 minutes followed by placement of cells in lysis solution at 4˚C for 1 hour. Electrophoresis was performed after washing the cells with PBS. Cells were placed in an electrophoresis tank for 30 mins after which ethidium bromide was added.

### Micronucleus assay

The IVM treatment was given to MDBK cells at (LD50, 2xLD50, LD50/2). Exactly 0.2 mL of media was obtained with the help of a pipette from wells of different doses that contained the MDBK cells. Centrifugation of cells was done at 2000 rpm for 5 min at 4°C. Supernatant was discarded and cells were transferred in 1 mL of PBS. Centrifugation was repeated at 1200 rpm for 5 min at 4°C. Supernatant was discarded and cells were suspended in PBS. A volume of 0.5 mL fixative II was added on cell pellet and centrifuged for 5 minutes at 4°C. Supernatant was discarded and fixative I was added on cell pellet which was again centrifuged for 5 mins after which the supernatant was again discarded. Then 0.1% KCL was added to the cell pellet. Suspended cells were placed on a cold wet slide which was dried at room temperature. Giemsa stain was added on the slide for 20 mins. Analysis for the presence of micronuclei was done under the compound microscope (Tokyo, Japan).

### RNA extraction

The RNA from MDBK cells was isolated by using the TRIzol method. About 100 μl of cells were taken and 300 μl TRIzol was added. Cells were mixed with 150 μl-200 μl chloroform and incubated at room temperature for 2 min. Afterwards, cells were centrifuged at 1200 G for 15 min at 4°C, and three layers were formed. The first layer (containing RNA) was sucked and an equal amount of isopropanol was added to it and incubated at room temperature for 10 min after mixing. Centrifugation was repeated at 12000 rpm for 10 min at 4°C and supernatant was discarded. A total volume of 1 mL of 75% ethanol was added and centrifuged at 7500 rpm for 5 min at 4°C. The supernatant was discarded and the pallet was air dried which was further resuspended in 20 μl of RNase-free water (DEPC-treated water). Finally, the sample was incubated for 10 min at 56°C in a water bath to facilitate solubilization.

### Reverse transcriptase PCR

Master mix was prepared by adding required amount of all reagents in 1.5 ml tube. 2 μl of mRNA was added in each labelled PCR tube and 18 μl of master mix was added to each tube containing different RNA samples. The tubes were then given a short spin and placed in Thermocycler. The PCR was done by using the standard PCR parameters.

### Gene expression analysis through RT-PCR

The RT-PCR was performed by using Thermo Fisher Scientific Maxima SYBR Green. The PCR master mix was prepared according to the given protocol. The total volume of RT-PCR master mix was 20μl which included 12.5 μl Maxima SYBR Green, 0.3 μM forward and reverse primers, less than 500 ng template DNA and nuclease-free water up to 20 μl. The PCR tubes were then placed on ice and template DNA along with master mix was added to the tube. After homogenizing the reagents with a short spin PCR was performed under optimized conditions which included denaturation at 95°C for 15s, annealing at 60°C for 30s and extension at 72°C for 30s for a total of 40 cycles. During the reaction, amplification was analyzed in real-time on the computer screen as a curve.

### Gene expression by Livak method

Gene expression analysis was done by measuring fold change i-e the expression ratio which demonstrates whether the gene is upgraded or downgraded. Positive values indicate that the gene is elevated whereas, negative values indicate down gradation of gene. Livak method (ΔΔCT) was used to estimate gene expression “Table 1” and the following formula was used to check the expression ratio of the *OGG1* target gene whereas, the *HPRT1* gene was used as a housekeeping gene

Formula: ΔCT(Test) = Ct (Target)—Ct (Housekeeping)

**Table 1:** Gene expression of *OGG1* and *HPRT1 gene* between control and treated samples.

		Gene Expression			
*OGG1* (Target Gene)			*HPRT1* (Housekeeping Gene)		
Doses	Control	Treated		Control	Treated
**LD 50**	0.04	9.67		0.007	4.76
**LD 50/2**	1.1	8.99		0.2	7.34
LD 50*2	0.0005	1.54		0.001	6.38

### Statistical analysis

The data (mean ± standard deviation) was analyzed using the Statistical Package for the Social Sciences (IBM® SPSS) program for Windows version 23. A one-way ANOVA with Tukey test (p≤0.05) was performed for statistical analysis. Furthermore, the effects of different doses were evaluated using Pearson correlation (p < 0.05) “Table 2”.

**Table 2:** Comparison of Mean and S.D of control (*GAPDH*) and target gene of interest (*OGG1* and *HPRT1)* of various IVM dose concentration.

Mean and S.D of Genes					
*OGG1* (Target Gene)				*HPRT1* (Housekeeping Gene)	
Doses	GADPH Control	Target		GADPH Control	Target
**LD 50**	22.6±1.2	36.6±0.9		21.6±0.9	34. 6±0.3
**LD 50/2**	19.7±0.3	26.3±3.1		16.3±1.0	37.3±3.4
LD 50*2	27.2±0.10	42.0±3.4		28.2±1.0	36.8±8.9

## Results

### MTT assay

**Fig 1:**
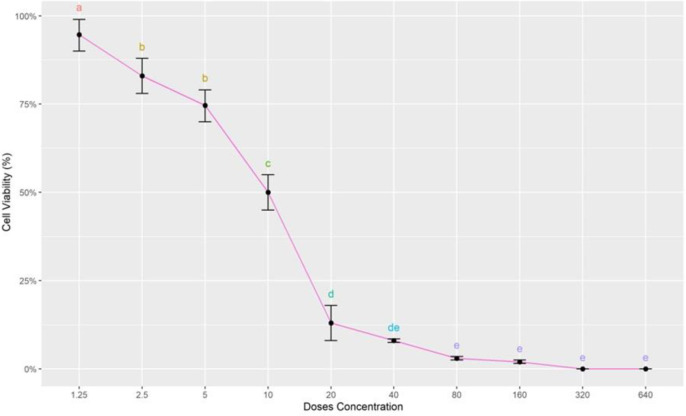
Cell viability (%) after the exposure of the series of concentration of ivermectin. X-axis showing the concentration of ivermectin and y-axis representing the Cell viability (%). Different alphabets showing the level of significance (p≤0.05).

During this experiment metabolically active cells reduced tetrazolium to formazan by the action of dehydrogenase enzymes. Viable cells were indicated by purple color whereas, dead cells were not stained. The percentage of cell viability decreased as the doses of IVM increased as shown in “Fig 1”. At LC50 dose of IVM the percentage of cell viability decreased up to 50%.

### Comet assay

**Fig 2:**
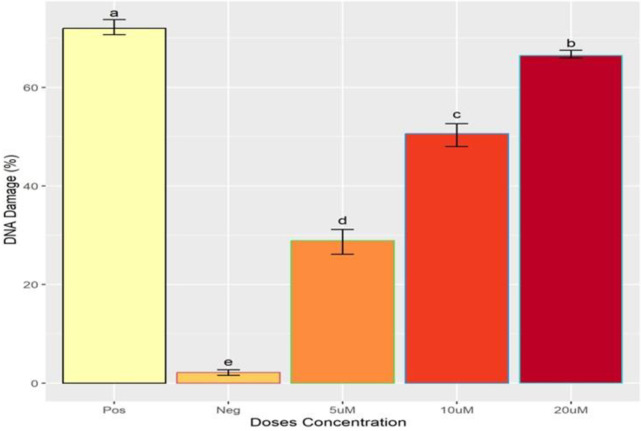
DNA damage findings after the exposure of Ivermectin. x-axis showing the concentration of ivermectin and y-axis representing the DNA damage. Different alphabets showing the level of significance (p≤0.05).

**Fig 3:**
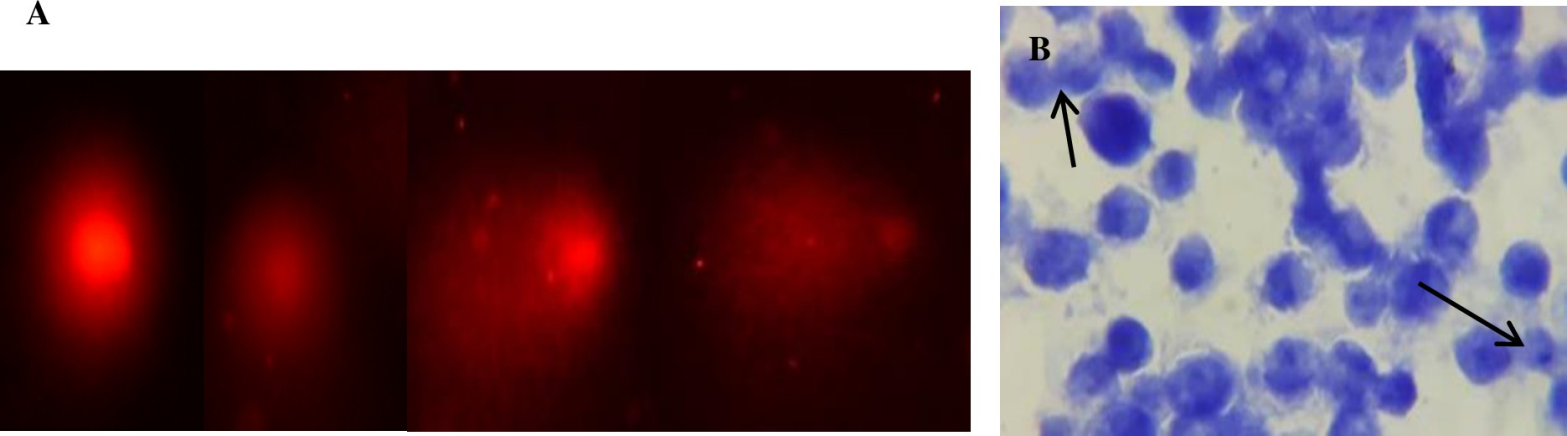
A. Comet formation observed under fluorescent microscope in MDBK cell line (Left side). B. Formation of Micronuclei in MDBK cell line (Right side). Scale bar 20 um.

**Fig 4:**
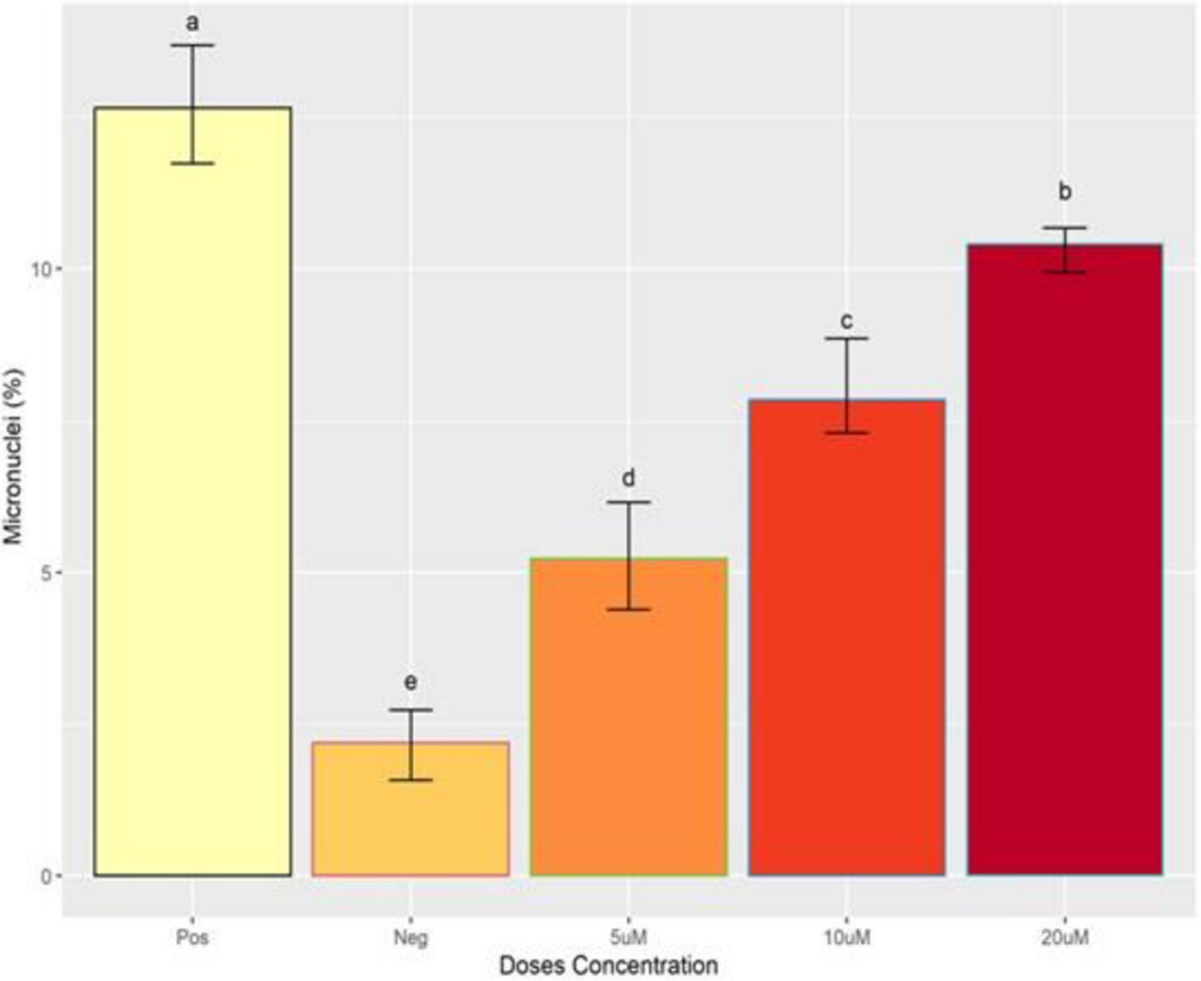
Micronuclei formation along with different doses of IVM concentration. x-axis showing the concentration of ivermectin and y-axis representing the DNA damage. Different alphabets showing the level of significance (p≤0.05).

Results of the current study demonstrate that the extent of DNA damage in MDBK cell line was dependent on dose of IVM. It was observed that a low dose of 5 μM caused less DNA damage compared to high dose of 20 μM as shown in “Fig 2”. The formation of the comet under a fluorescent microscope is shown in “Figs 3 and 4A".

### Micronucleus assay

Micronuclei formation shows genotoxicity in MDBK cell line after treatment with different concentrations of IVM which is due to its genotoxic nature “Fig 4B”. Micronuclei formation was found to be directly proportional to the concentration of IVM as shown in “Fig 3”.

### RNA extraction

A total of 8μg of pure RNA was extracted from a plate with 6 wells. Different concentration of RNA was extracted from each well containing MDBK cells. The well containing MDBK cells with a 5 μM dose yielded a concentration of 1575.8 RNA whereas, the well comprising of MDBK cells with a 10 μM dose yielded a concentration of 1385.2 RNA. It was also noted that the well containing MDBK cells with a 20 μM dose yielded an 816.4 RNA concentration.

### Reverse transcriptase PCR

RNA isolated from cells treated with 20 μM of IVM resulted in a lower amount of cDNA compared to cells treated with 5 μM of IVM. This suggests that high dose of IVM causes more DNA damage than low dose.

### Gene expression analysis by qRT-PCR

In quantitative PCR (qPCR) assay a higher cycle threshold (Ct) value indicates lower gene expression while a lower Ct value indicates higher expression. Based on the results of this experiment, the Ct values for 5 μM IVM, 10 μM IVM and 20 μM IVM were 18.72, 20.04 and 22.01 respectively, demonstrating that different doses of IVM directly affect Ct values. This suggests that cells treated with higher doses of IVM have higher gene expression of *OGG1* while those treated with lower doses have lower expression of *OGG1*. These findings support the hypothesis of this study that the expression of the *OGG1* gene increases as the dose of IVM increases.

## Discussion

The IVM is derived from avermectin B which is an antiparasitic drug that comes from bacterium Streptomyces. It is effective against worms and arthropods and is useful in treating parasitic infestation related to both humans and domestic animals. However, its toxic nature can cause development of micronuclei [14]. The IVM works by binding to ligand-gated ion channel receptors such as glutamate, GABA and glycine. These receptors are responsible for motility, reproduction and feeding actions in the parasite. In vitro studies have shown that IVM has antiviral properties at very high doses [15]. The IVM is a proven antimicrobial and antiviral agent known for prophylaxis or treatment that prevents re-occurring of disease [16,17] and is used widely for treatment of endoparasites and ectoparasitic infections in animals as well as in humans [18]. Many nematodes have become resistant to IVM due to its excessive use, under dosage genetic defense of pests and many other factors [19].

In this study the effect of IVM on MDBK cell viability was determined using MTT assay. The color intensity was used to measure cell viability which decreased with high doses of IVM. A lethal dose (LD50) was selected at which only 50% of cells were viable and 50% were killed. Live cells stained purple whereas, dead cells remained colorless. The color intensity was measured by ELISA plate reader at 630nm. The optical density at different doses provided the percentage of viable cells. Our analysis showed that cytotoxicity induced by IVM in MDBK cells decreased viability up to 75% with an increase in its concentrations up to 20 μM. This indicates that higher doses of IVM are more hazardous than lower doses.

Exposure of MDBK cells to IVM lead to genotoxicity by inducing DNA damage. During comet assay it was observed that the length of the comet (tail) was dependent on the dose of IVM. Cells treated with 10 μM dose of IVM exhibited a clear and long comet (tail) as compared to cells treated with 5 μM dose of IVM. This suggests that 10 μM of IVM caused more DNA damage as compared to 5 μM. Similarly, 20 μM of IVM caused more damage than the 10 μM dose. Findings of this study suggest that higher doses of IVM cause more DNA damage to cells than lower doses. Furthermore, micronucleus assay was also performed to represent the genotoxicity induced by IVM in MDBK cells [14,20]. After treatment with IVM small membrane-bounded nuclei called micronuclei are formed inside MDBK cells indicating DNA damage. The formation of micronuclei was found to be dependent on the dose concentration as our results demonstrate that 10 μM dose of IVM had more micronuclei than a 5 μM dose. Moreover, MDBK cells treated with 20 μM dose of IVM had more micronuclei compared to 10 μM. These results are consistent with the findings of De sousa *et al*. [21] who reported that high concentration of IVM is more lethal compared to low concentration.

The cDNA was used to determine the expression of the *OGG1* gene through qPCR expression. The *OGG1* gene is known to be expressed in response to genotoxicity [22] induced by IVM in MDBK cells. The expression levels of the *OGG1* gene were found to increase with an increase in the dose of IVM. Higher expression levels were observed in cells treated with 20 μM compared to cells treated with 5 μM.

Upregulation refers to an increase in the expression or activity of a gene. In this study the *OGG1* gene demonstrated a significant increase in expression by a factor of 6930.97-fold. The *OGG1* gene is responsible for DNA repair and plays a critical role in repairing a specific type of DNA damage known as 8-oxoguanine. The *OGG1* assists cells in repairing DNA damage and reduces the risk of genetic mutations. The damage of DNA can occur from various sources which include exposure to toxins, radiations or chemicals. The reason for high-fold gene expression is an elevated level of DNA damage in MDBK cells due to the administration of a higher dose of IVM. The upregulation of the *OGG1* gene indicates that the cells are trying to enhance their DNA repair mechanisms to counter the damage caused by high doses of IVM. An increase in *OGG1* gene expression signifies that MDBK cells are responding to high levels of DNA damage by producing more *OGG1* protein. This is a protective mechanism for repairing DNA and preserving genomic integrity which indicates that the cells are actively working to mitigate the effect of DNA damage caused by IVM.

Hoti *et al*. [23] discussed the cytotoxic effects on a colon cancer cell line HCT-116 using MTT assay analysis. According to IVM exhibits significant anti-cancerous activity and enhances the tubulin polymerization mechanism. However, our findings contradict his study. Ivermectin has been reported to directly affects target cells along with stopping unnecessary cell proliferation through apoptosis triggering. Results from the findings of De sousa *et al*. [21] reveal that IVM causes carcinogenic and cytotoxic effects after chronic exposure in an *in vivo* analysis on *Drosophila melanogaster* and *Tradescantia pallida*. Our results are in line with the aforementioned study. Furthermore, El-Saber *et al*. [24] analyzed the genotoxic effect of IVM by examining its neurotoxicity at higher doses indicating a specific safe zone of this chemical. However, higher doses can be lethal depending on the substrate or target [5].

The use of IVM caused a genotoxic effect as it led to significant changes in the expression of 36 proteins in the gilthead sea bream liver protein profile, which was indicated by the Difference Gel Electrophoresis Technology (DIGE) [25]. However, IVM did not exhibited genotoxic effects in adult zebrafish. A multilevel assessment of IVM effects was analyzed using different zebrafish life stages, which is in contrast to our findings [12]. Although behavioral responses including lethargy, occurred in all-life stages and many altered biochemical responses were observed showing that there are cytotoxic and basically genotoxic effects present that are due to higher concentration or species difference compared to our experiment [12,26,27].

## Conclusion

Nexus to the above results of this study suggest that Ivermectin (IVM) has significant cytotoxic and genotoxic effects on the Madin-Darby-Bovine-Kidney (MDBK) cell line particularly at higher concentrations. The genotoxic effects were evident from the increased expression level of the *OGG1* gene in the MDBK cell line which concludes that IVM cause DNA damage in MDBK cells. Furthermore, it is recommended that studies related to the toxic effects of IVM at molecular level should be conducted to combat the hazardous effects caused by IVM.

## Supporting information

S1 Data (CSV)

S2 Data (XLSX)

S3 Data (CSV)
